# Managing Coronary Thrombus in the Cath Lab During PCI

**DOI:** 10.2174/157340312803217166

**Published:** 2012-08

**Authors:** John A Ambrose

The last two articles specifically address the question of therapeutic maneuvers for dealing with intracoronary thrombus during PCI. Either the thrombus is removed by aspiration or manual thrombectomy or embolic protection devices are deployed to prevent downstream embolization. Are there any other methods in selected cases? 

No one in the symposium has considered thrombolytic therapy as a method for dissolving thrombus during PCI. In 1994, our group published the Thrombolyisis and Angioplasty in UnStable Angina (TAUSA) trial which found that routine intra coronary thrombolytic therapy immediately before and after balloon angioplasty for unstable angina was detrimental [1]. However, that was prior to stenting. Other studies indicated that in selected cases, an infusion of IC thrombolytics could be effective for dissolving large intracoronary thrombi albeit at the risk of increasing bleeding complications [2, 3]. 

In the following case done in 2012, a patient with an ectatic, tortuous right coronary artery with an intra coronary thrombus distal to a severe complex lesion underwent intervention after a NSTEMI. The wire could not cross the lesion, so mechanical and aspiration thrombectomy were not an option. The patient was treated with IV integrilin overnight and 15milligrams of tissue plasminogen activator (t-PA) were infused into the ostium of the right coronary artery over 20 minutes. The sheath was left in place and the patient returned the next day. At that time, a Kinetics-Plus wire was successfully placed across the lesion and a 5.0mm bare metal stenting was deployed with excellent results. 

The t-PA/integrilin combination had dissolved the thrombus distal to the lesion (Fig. **1-3**). The patient tolerated the procedure without significant bleeding. 

These results underscore the possibility in very selected cases of utilizing this combination approach for large intra coronary thrombi in an open artery when other approaches are not possible. Whether integrilin alone with an anti thrombin would have produced the same results is unknown but this thrombus was likely fibrin rich and these are quite sensitive to small doses (10 to 15mgs) of a locally delivered thrombolytic. 

## Figures and Tables

**Fig. (1):**
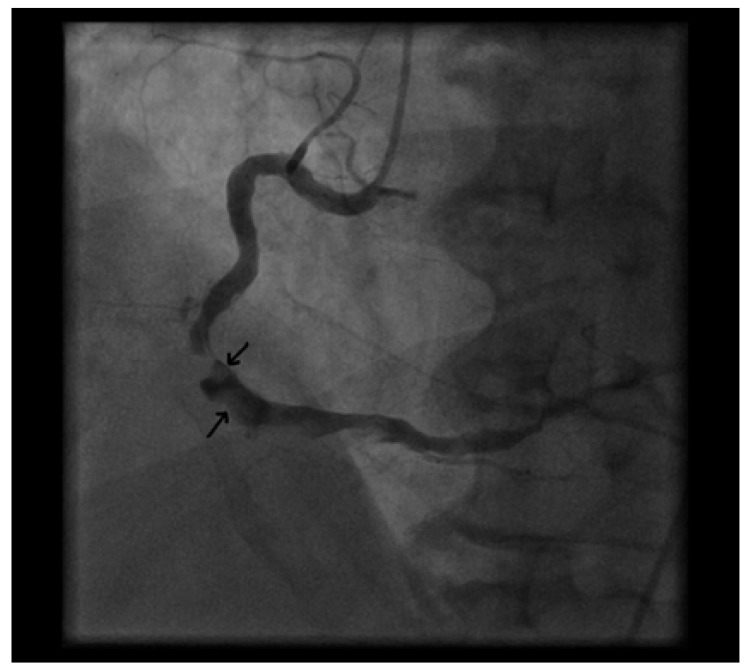
Right coronary artery in the LAO position showing the severe lesion at the crux with post lesion thrombus (arrows) pre IC thrombolytic and IV integrilin.

**Fig. (2):**
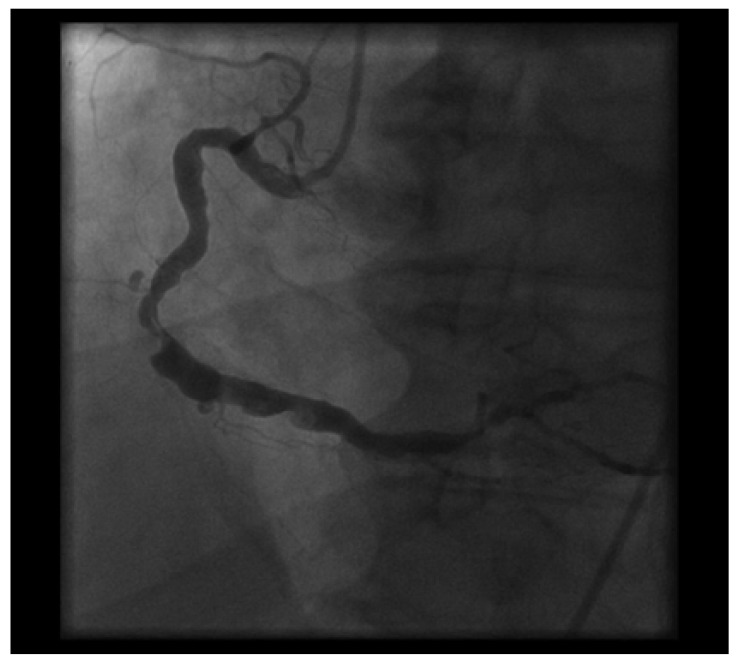
The same projection as Figure 1 injected 24 hours later showing near complete or complete resolution of post lesion thrombus.

**Fig. (3):**
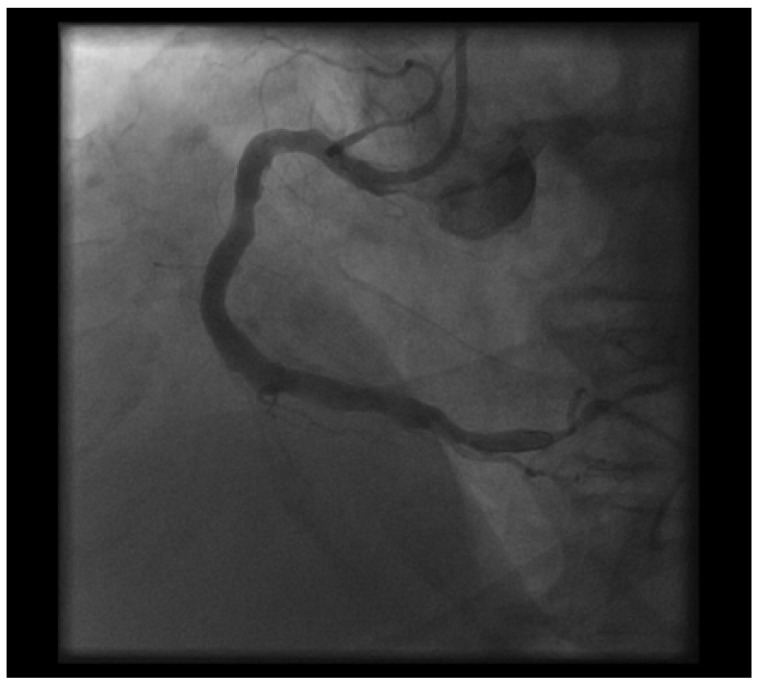
Post stenting and post balloon dilatation showing excellent result.
